# Who has sheds? Exploring practices and determinants of overnight housing for backyard poultry in rural Bangladesh to inform an intervention to limit exposure to poultry and poultry feces

**DOI:** 10.1371/journal.pgph.0004929

**Published:** 2025-07-22

**Authors:** Elizabeth D. Thomas, Laura H. Kwong, Nadia Ali Rimi, Ireen Sultana Shanta, Mohammad Rofi Uddin, Shifat Khan, Salma Akter, SM Monirul Ahasan, Mahbub-Ul Alam, Mahbubur Rahman, Tarique Md. Nurul Huda, Peter J. Winch

**Affiliations:** 1 Department of International Health, Johns Hopkins Bloomberg School of Public Health, Baltimore, Maryland, United States of America; 2 Department of Environmental Health Sciences, University of California, Berkeley, Berkeley, California, United States of America; 3 Emerging Infections, Infectious Diseases Division, icddr, b, Dhaka, Bangladesh; 4 Environmental Health and WASH, Health Systems and Population Studies Division, icddr, b, Dhaka, Bangladesh; 5 Global Health and Migration Unit, Department of Women’s and Children’s Health, Uppsala University, Uppsala, Sweden; 6 Department of Public Health, College of Applied Medical Sciences, Qassim University, Buraydah, Saudi Arabia; PLOS: Public Library of Science, UNITED STATES OF AMERICA

## Abstract

Backyard poultry-rearing contributes to income and food security for rural households in low- and middle-income countries. However, poultry are often kept inside the household dwelling at night, posing health risks to the people raising them. Housing poultry separately from the dwelling overnight is a potential intervention to limit exposure to poultry. The aim of this study was to describe practices and determinants of overnight poultry housing in rural Bangladesh as formative research for an intervention to separate young children from poultry and poultry feces. We conducted 19 transect walks in villages across Bangladesh to document overnight housing practices among backyard poultry raisers. We then conducted 27 semi-structured interviews to explore poultry-raising practices, including housing types and materials identified during transect walks. We found overnight poultry housing both inside and separate from the dwelling and found that most poultry raisers who kept their birds separate from the dwelling overnight did so in courtyard sheds. There was a preference and willingness to house birds outside, provided a shed was available, although overnight housing practices fluctuated. Having a shed was a function of household resources, including availability and access to materials and skilled labor, available physical space, area- and village-wide trends, and the preferences and concerns of poultry raisers. We recommend that future studies measuring human exposure to poultry and poultry feces assess exposure prospectively and at regular intervals to capture variations in housing practice, and include assessments of poultry housing hygiene practices. The promotion of sheds for overnight poultry housing may be an acceptable intervention approach in this setting, though programs will need to make recommendations for housing that address the risk of zoonotic disease transmission and accommodate the preferences and constraints of poultry raisers over a one-size-fits-all approach.

## Introduction

Backyard poultry-rearing contributes to income and food security for most rural households in low- and middle-income countries (LMICs) [1–3]. The benefits of raising poultry can be obtained with relatively low input, making it an accessible asset for poor households [4]. Poultry meat and eggs can contribute to animal source food consumption [5–7], or can be sold for cash to pay for school materials and fees or medicine [2]. Backyard poultry are most often reared by women, which can increase women’s access to and control over household assets [8,9]. However, backyard poultry production often includes few biosecurity measures and can therefore pose health risks for humans [2,10–15]. Public health concerns from backyard poultry include increased risk for diarrheal disease and enteric infections [11,16–21], respiratory infections including highly pathogenic avian influenza A (H5N1) [22], and other zoonotic infections and zoopotentiation [23,24].

In backyard poultry production systems in LMICs, birds are typically left to scavenge for food during the day, and at night are commonly kept inside the household dwelling where people sleep [2,13,25]. Studies from Bangladesh [18], Ethiopia [19], and Tanzania [26] suggest an increased risk of adverse health outcomes for young children, including diarrhea and growth faltering, when poultry are kept within the dwelling overnight. In Ethiopia, Headey and Hirvonen (2016) found that poultry ownership alone was positively associated with child growth but that keeping poultry inside the dwelling overnight was negatively associated with child growth [19]. In Bangladesh, corralling animals, including poultry, inside the child’s sleeping space at night was associated with a more than two times higher odds of stunting as well as elevated fecal markers of environmental enteric dysfunction [18], a subclinical gut disorder linked to poor child growth and other adverse health and economic outcomes [27–30]. In Tanzania, housing chickens inside any room of the dwelling overnight was associated with an increased risk of diarrhea for children [26]. One hypothesis for these associations is that housing birds inside the dwelling overnight and inadequate hygiene elevates contacts with poultry feces, increasing the risk of exposure to enteric pathogens commonly carried by poultry, such as *Campylobacter* spp*.* and *Salmonella* spp. [14,19,31–33]. Housing poultry inside the living space with people has also been cited as a risk factor for exposure to Influenza A virus subtype H5N1 [34,35]. This body of research calls for interventions to better separate poultry from humans.

Housing poultry separately from the dwelling overnight is a potential intervention to limit human exposure. In Bangladesh, where the majority of rural households raise backyard poultry [36], a nationally representative study found that over one-third of respondents (N = 2489) kept their chickens inside their homes with household members at night [37]. Another study (N = 184) found that poultry were kept at night in the bedroom (46%), on the veranda (26%), in the yard (15%), or some combination of these locations (5%) [9]. Other studies in Bangladesh have documented similar variation in poultry housing location [18,38,39] as well as variation in housing design and material [38–40]. In these studies, it was not always clear why some households kept their poultry inside the dwelling at night while others did not.

Among poultry raisers in Bangladesh and elsewhere, a commonly cited reason for keeping birds inside the dwelling overnight is concern for theft or predation [9,13,37]. However, overnight housing practices vary both regionally and within the same community [9,37–40], suggesting that factors beyond theft and predation likely influence where and how people house their poultry. These findings indicate a need to further explore the patterns and determinants of overnight housing for backyard poultry—such research is critical for developing interventions that aim to leverage existing overnight housing practices to better separate poultry from humans. In this study, we describe practices and determinants of overnight poultry housing in rural communities across Bangladesh as part of formative research for an intervention to separate young children from poultry and poultry feces, with implications for exposure assessment and intervention programs.

## Methods

### Study site

This study was nested in a larger formative research study at icddr,b that sought to identify a range of existing practices in rural poultry-raising homes across Bangladesh that could be leveraged to separate children from poultry and poultry feces [41].

In Bangladesh, divisions are the largest administrative unit—there are eight total. Each division is divided into administrative and local government units; unions, which are made up of several villages, are the smallest rural administrative unit. Within villages, related households are typically organized into compounds, where households often share a latrine, water source, and certain spaces, such as a courtyard or pond, but each household has its own dwelling and cooking space. Compounds are rarely fully enclosed and often have open entryways, facilitating the movement of people and domestic animals between compounds and other spaces in the village. Several studies conducted in this setting have documented the challenging hygiene conditions that increase young children’s risks for exposure to enteric pathogens, including through exposure to domestic animals [42–47].

### Transect walks

#### Data collection.

From 26 January to 25 March 2019, trained field researchers conducted transect walks (n = 19) in teams to identify practices that could facilitate separation of children from poultry and poultry feces. A transect walk is a systematic walk along a defined path in an area, often a rural village, during which researchers document resources and practices in collaboration with community members [48].

The study team purposefully selected unions with a high proportion of poultry-raising households, based on prior research and experiential knowledge [9,49,50]; between two and five unions were selected for transect walks from each of the eight divisions in Bangladesh, and one village was selected per union.

In each village, field researchers facilitated a brief group discussion with a convenience sample of community members to introduce the study objectives and establish the village boundary. During discussions, field researchers asked about poultry-raising practices, including housing. Then, field researchers walked in one direction until reaching a geographical endpoint (e.g., boundary, body of water), visiting 10–15 households along their route. Field researchers purposefully looked for households or compounds that had poultry, at least one child under five years of age, and any type of poultry confinement or feces management strategy. Field researchers spoke informally with any poultry raiser present, took notes on the number and types of poultry owned and poultry-housing and other poultry-raising practices, and took photographs of practices at most households. Given the main study objective, attention was given to documenting a range of strategies that could be leveraged to separate children and poultry. A total of 222 households were visited across all divisions.

#### Analysis.

Field researchers extracted data from field notes and charted them in Microsoft Excel. Members of the study team then compiled and categorized all transect walk photographs according to their primary purpose as described by the poultry raiser (e.g., overnight housing). Photos of any poultry housing were also categorized by their material components (e.g., categorized by the material the majority of the shed was made of (primary material)). For this study, one author (ET) also manually coded photographs and field notes for patterns in the design, material, and location of overnight poultry housing, where data were available; data for 164 sheds documented during transect walks were included in this analysis (S1 Data).

### Semi-structured interviews

#### Data collection.

**Fig 1:**
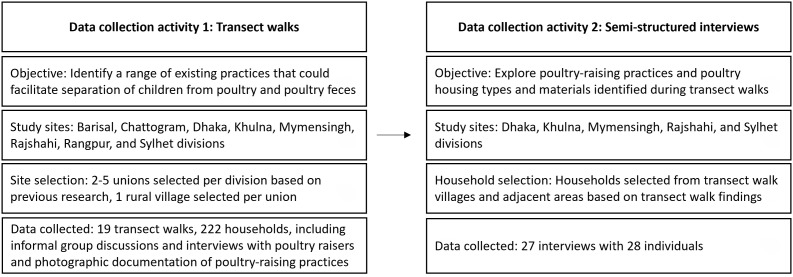
Research sites and activities.

The study team purposefully selected a sub-set of households (n = 15) from transect walk villages from five divisions to interview about the range of poultry housing types and materials documented (Fig 1). They then interviewed other households (n = 10) in the same or nearby villages as early findings indicated new areas of inquiry, such as the need to further explore the practice of keeping poultry inside the dwelling overnight. Five of those interviews were conducted in Mymensingh division after it was selected for the parent study trial (Table 1) [41]. Two beneficiaries of a program organized by the Voluntary Association for Rural Development (VARD) were also interviewed. Beneficiaries of the VARD program received training and financial support for building poultry housing. Interviews took place between 03 April and 26 August 2019.

**Table 1:** Data collection activities and semi-structured interview participant characteristics.

Transect walks	N or Mean (Range)
No. of transect walks completed	19
No. of transect walks per administrative division^a^	Villages, Households
Barisal	2, 22
Chattogram	2, 24
Dhaka	5, 64
Khulna	2, 24
Mymensingh	2, 20
Rajshahi	2, 24
Rangpur	2, 24
Sylhet	2, 20
**Semi-structured interviews**	
No. interviews completed	27
No. interviews per administrative division	
Dhaka	8
Khulna	5
Mymensingh	8
Rajshahi	4
Sylhet	2
No. of participants interviewed^b^	28
Female	26
Participant age^c^	33 (20-51)
Participant educational attainment^d^	
Not available	8
No formal education	6
Primary	8
Secondary or higher	6
Participant dwelling type^c^	
Mud	3
Corrugated iron and mud	4
Corrugated iron	9
Pucca or semi-pucca (brick and plaster)	7
Poultry raised by household^e^^,^^f^^,^^g^	
Chickens (n = 24)	8.5 (1-25)
Ducks (n = 15)	4.5 (1-16)
Pigeons (n = 3)	8.7 (4-14)
Other (n = 5)	3.6 (2-6)
Location of overnight poultry housing for household^e^	
Separate from the household dwelling	19
Inside the household dwelling	5
Both inside and separate from the household dwelling	3

^a^ 2–5 unions selected per division based on previous research, 1 village selected per union for transect walk.

^b^ 27 interviews conducted with 28 participants (one interview was conducted with two female poultry raisers from the same household).

^c^ 4 missing values for participant age and dwelling type.

^d^ 8 missing values for participant educational attainment.

^e^ Values provided for household unit, not individual participant.

^f^ Means calculated only for households raising type of poultry.

^g^ Households could raise multiple types of poultry.

**Fig 2:**
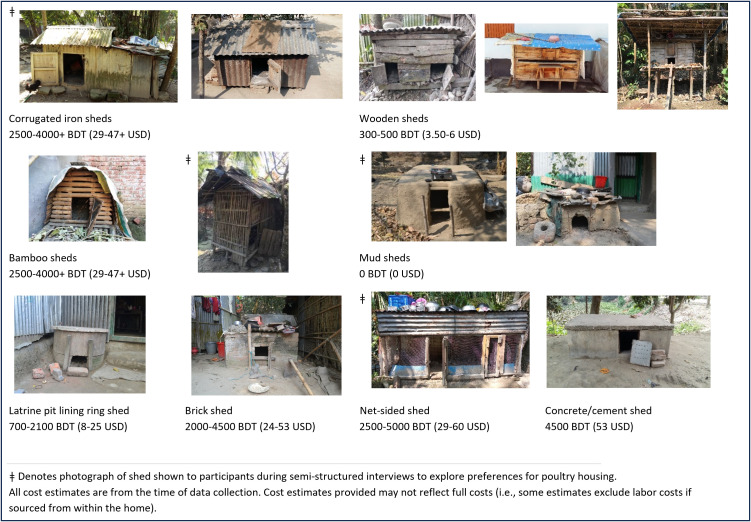
Examples illustrating variation in design and materials for sheds documented during transect walks.

The study team developed an interview guide broadly informed by the Integrated Behavioral Model for Water, Sanitation, and Hygiene (IBM-WASH) [51]. IBM-WASH was chosen as a framework to inform our approach to exploring poultry-raising practices in this setting because it acknowledges contextual (e.g., seasonal variation) and technology (e.g., strengths and weaknesses of housing) factors at multiple levels, including the individual (e.g., poultry raiser practices and preferences), household (e.g., roles and responsibilities), and community (e.g., common practices). Field researchers trained in qualitative data collection conducted the interviews in Bengali, using the interview guide, which included questions on poultry-raising practices, poultry-housing practices, and preferences for poultry housing. To explore preferences for poultry housing, in the majority of interviews, participants were shown photographs of four sheds identified during transect walks and asked about their strengths and weaknesses (Fig 2). The four shed types were selected because they represented a range of materials (mud, bamboo, corrugated iron, and concrete/cement and netting), price points (0–5000 BDT), and other features (e.g., variation in ventilation) of housing.

The primary interview participant was the individual responsible for poultry in the household, though other household members sometimes joined to provide supporting information, such as details on the cost of poultry housing. Interviews were audio-recorded with consent.

#### Analysis.

Interviewers and supervisors completed comprehensive debriefs for all interviews to facilitate concurrent analysis and guide future iterations of data collection [52]; they then compiled and summarized debriefs by topic as outlined in the interview guide. One author (ET) reviewed all summaries and requested clarification from the interviewer if needed, who then expanded summaries based on audio recordings. Transcription of all audio recordings was completed in Bengali, with select translation into English.

One author (ET) conducted the analysis for this study in four iterative rounds, which included: 1) Reviewing data during data collection and debriefing; 2) Reviewing all debriefing sessions and organizing data into concept maps as part of initial coding [53]; 3) Applying deductive and inductive codes to all debriefs and select translations using ATLAS.ti, and writing analytic memos throughout the coding process [54]; and 4) In multiple rounds, reviewing and manually grouping, refining, and expanding coded data to identify categories and themes, and organizing findings informed by the levels and dimensions of the IBM-WASH framework to facilitate interpretation [51]. Study objectives and the organization of the interview guide informed deductive codes.

### Ethical considerations

Field researcher teams obtained verbal consent from study participants for all transect walk activities; verbal consent was selected due to these being chance, brief encounters with participants. Field researchers obtained written consent from all primary semi-structured interview participants. The Ethical Review Committee of icddr,b reviewed and approved this research protocol (PR-18087).

## Results

### Transect walks

**Fig 3:**
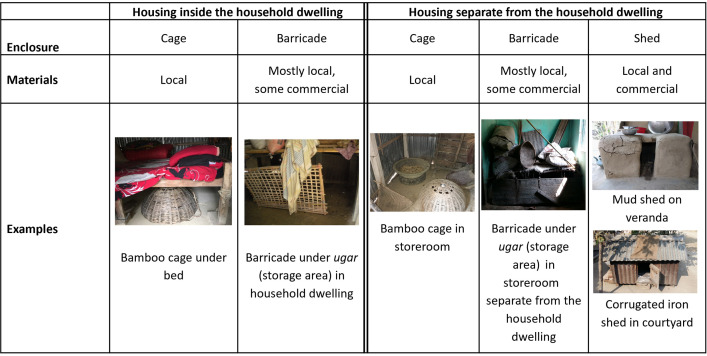
Poultry housing identified inside the household dwelling and separate from the household dwelling.

We found various examples of overnight poultry housing inside the dwelling, in the compound courtyard in varying distances to the dwelling, on household verandas, in storerooms, and in kitchens (both attached and separate from the dwelling) (Fig 3). Some poultry housing was shared with other domestic animals (e.g., goats or cattle). The poultry housing identified was made from a mix of local and commercial materials.

Across all divisions, a common practice was to house poultry in bamboo cages, which were often placed under a bed or agricultural storage area (*ugar*) inside the dwelling or in a separate structure (e.g., cowshed). Another type of overnight housing described was barricades made from mud, bamboo, plastic, tin, or other materials, used to enclose poultry under an *ugar* or furniture, or in corners inside the dwelling or a separate structure (Fig 3).

We also documented “sheds”, house-like enclosures for poultry that had walls, a roof, and a door with either single or multiple compartments, made from a variety of materials and of variable design, size, and quality, with some possible regional trends (Table 2, Fig 2, S1 Data). Sheds were found most often in the compound courtyard, or in another space separate from the dwelling (e.g., veranda or storeroom).

**Table 2:** Primary and secondary materials of sheds in each division.

Primary material of sheds (n = 164)^a^								
	Division							
	Barisal (n = 11)	Chattogram (n = 24)	Dhaka (n = 52)	Khulna (n = 21)	Mymensingh (n = 11)	Rajshahi (n = 24)	Rangpur (n = 15)	Sylhet (n = 6)
Bamboo	0	0	0	1	0	5	5	0
Brick	0	2	8	2	2	7	1	0
Concrete/cement	0	4	3	2	0	0	3	0
Mud	0	0	3	7	0	4	1	0
Net	0	1	0	1	0	0	0	1
Latrine pit lining ring (cement)	0	8	21	0	0	0	3	0
Steel	0	0	1	0	0	0	1	0
Corrugated iron	0	4	13	0	8	1	1	4
Wood	11	5	3	8	1	7	0	1
**Secondary material of sheds (n = 119)** ^b^								
	Barisal (n = 7)	Chattogram (n = 20)	Dhaka (n = 27)	Khulna (n = 19)	Mymensingh (n = 10)	Rajshahi (n = 21)	Rangpur (n = 9)	Sylhet (n = 6)
Bamboo	3	1	0	3	0	1	2	0
Brick	0	6	2	1	0	1	0	0
Concrete/cement	0	1	3	1	1	1	1	0
Mud	0	0	0	1	0	0	0	0
Net	0	1	0	2	1	1	0	1
Latrine pit lining ring (cement)	0	1	0	0	0	0	0	0
Steel	0	1	0	0	0	1	0	0
Corrugated iron	4	6	8	2	1	9	1	2
Wood	0	3	11	2	6	4	0	2
Other (e.g., plastic)	0	0	3	7	1	3	5	1

^a^ The primary material of the shed is the material the majority of the shed is made of (e.g., all walls); Data available for 164 sheds.

^b^ The secondary material of the shed is the second most included material of the shed (e.g., roof, floor), if recorded or visible; Data available for 119 sheds.

Some materials were more commonly documented for housing across regions, like wood and corrugated iron, while others were less common, like bamboo and steel (Table 2). Mostly in Dhaka, as well as in Chattogram and Rangpur, we found cement latrine pit lining rings and covers modified to serve as poultry housing in the courtyard, storeroom, or cowshed. Regardless of design, material, and location, we found that poultry housing could be called by the same name. Common names for overnight poultry housing, including sheds, barricades, and cages, included *khoar/khoara* (barn/corral), *ghor* (house/room), and *basa* (house/home).

For this study, we distinguish between two main categories of overnight poultry housing (housing inside the dwelling and housing separate from the dwelling) and three types of enclosures (cages, barricades, and sheds) as described above and in Fig 2. While we found cages and barricades both inside and separate from the dwelling, we only found sheds in locations separate from the dwelling. Sheds were the only type of overnight enclosure for poultry found in courtyards.

In the majority of villages, we documented variation in location and type of housing in both field notes and photographs. However, most photographs of barricades were from Sylhet and Mymensingh divisions, as were most photographs of housing inside the dwelling. The following excerpts from field notes also suggest possible area trends in location and type of overnight poultry housing.

All the households [in this village] follow almost the same strategy to confine backyard poultry. They usually use sheds for keeping both chickens and ducks at night, which are locally called *kuthi*.

- Field notes excerpt, Rajshahi Division, 01-March-2019

They (participants) said more than 80 percent of the households [in this village] raise their poultry in their living room under the *ugar* (storage area) or bed.

- Field notes excerpt, Sylhet Division, 07-March-2019

### Semi-structured interviews

We conducted 27 semi-structured interviews. Most interviews (22/27) were with poultry raisers who had at least some overnight poultry housing separate from the dwelling. Five interviews (all in Mymensingh division) were conducted with poultry raisers who exclusively had overnight poultry housing inside their dwelling (Table 1). Participants’ poultry housing was made of a range of materials (S2 Data). The households and compounds where interview participants lived were typical of rural Bangladesh, and participant dwelling type included a range of mud, corrugated iron, and *pucca* (i.e., built from solid materials, like brick and plaster) dwellings. Participant dwelling type was considered a proxy for household socioeconomic status, as household income data were not collected. A previous study did not find differences with poultry housing patterns across socioeconomic conditions [9]. Among our interview participants, poultry housing type and material varied across participant dwelling type.

Interview participants had flock sizes ranging from 2-30 birds and raised mostly indigenous (*deshi*) breeds of chickens and ducks, known collectively as *hash-murghi* (poultry). A few also raised turkeys, pigeons, and geese. Participants commonly referenced recent changes to their flock composition; many mentioned recent losses of birds due to disease or predator attacks.

In the majority of interviews, poultry were described as owned and the responsibility of one household member, who was responsible for letting birds in and out of enclosures, feeding them, giving them medicine when needed, cleaning up after them, and watching over them during the day. As anticipated for this setting [9,37], in almost all households, the person primarily responsible for poultry was female. A few female participants said that male household members helped with poultry raising by buying medicine or food. A couple of participants mentioned that their husbands were not supportive of poultry raising. Additional participant information is provided in Table 1 and S2 Data.

#### Poultry-housing practices.

Most poultry raisers said that during the day, they let their birds roam freely for food within and beyond the compound, but also practiced temporary confinement to feed birds, protect them from predators, or keep them away from seedlings. In the evening, they called their poultry back to the compound, or the birds returned on their own, and were housed in their overnight enclosures, where they stayed until the next morning. No poultry raisers allowed their chickens or ducks to remain loose outside in the compound or village at night; one participant said she did sometimes leave her geese loose within her compound at night, as there was a brick wall around it.

Regardless of if they had poultry housing separate from their dwelling, a third of participants mentioned keeping laying hens and recently hatched chicks inside the dwelling overnight for short periods of time (1–3 weeks) to protect them from predators and other birds in the flock.

For the participants that reared multiple species of poultry, like ducks and chickens, some housed birds together at night, while others kept birds separately (fully or partially). For example, some housed ducks in one cage and chickens in another, though both were under the same *ugar*. Others housed different species in different compartments within the same shed, or, like some of the examples in the following sections, housed some birds inside their dwelling at night, and others separate from the dwelling. Participants gave several reasons for needing to separate birds, including preventing conflict between them and limiting disease. However, not all participants felt that different species needed to be separated.

#### Overnight poultry housing separate from the household dwelling.

For those interviewed with poultry housing separate from the dwelling, all had sheds in the compound courtyard. Most poultry raisers said their sheds were built by a male household member or a skilled worker (e.g., carpenter), though one participant said her mud-made shed was built by a female household member and another said she herself built the shed with her husband and son (S2 Data). Many participants reported that their sheds were built from materials they had on hand, such as leftover from a home renovation, or built from materials cultivated by the household (e.g., bamboo), or purchased from the market. Purchasing all materials with the intention of building a shed was not common. A few participants said they had selected the material for their shed due to personal preference, because it was common in their area, or because it would provide the best safety against predators or thieves. Decisions about shed type were made by a balance of the poultry raiser, male household member that was not the poultry raiser, or both.

*We just like the wooden coop, so we got it… It is easy to clean. We don’t use the clay (mud) coop because snakes can get in those coops.*

- Interview #23, Rajshahi Division, Female, 7 chickens and 2 ducks

*We bought this [latrine pit lining ring shed] because many people in this village are using the same shed. We had a mud-made shed, and then we had a small tin-made shed. We used that tin-made shed for 20–30 years, but the lower deck had rotted, and it was difficult to open the door.*

- Interview #6, Dhaka Division, Female, 2 chickens

For sheds where materials and labor had been sourced from within the household, the cost was sometimes difficult to recall or estimate, but the overall range of estimates was 0–5000 BDT (0–60 USD) (S2 Data). A cost estimate of 0 BDT was given for a mud shed, which did not include labor costs—the poultry raiser said the shed took 7 days to build, spending 2–3 hours per day on it. Up to 5000 BDT was given for a shed made with commercial materials (e.g., net-sided shed) (Fig 2). Latrine pit lining rings and covers were purchased for 700–2100 BDT (8–25 USD) and then assembled at home to make a shed. Three of the four poultry raisers interviewed from Rajshahi said they purchased prefabricated wooden sheds from the market for 200–400 BDT (2.50–5 USD)—low-cost compared to other shed types. The cost estimates for sheds are included in Fig 2. All cost estimates are from the time of data collection.

As seen during transect walks, sheds belonging to interview participants were in varying distances to the dwelling. One reason mentioned for keeping a shed close to the dwelling was to allow poultry raisers to keep an eye on birds in case thieves or predators came during the night. Others said the location was chosen because it was the only space available. One participant said she had selected the location for her shed because it was visible to her but far enough from her mother-in-law’s dwelling; her mother-in-law had previously complained about the smell coming from the shed. Like decisions about the shed design, shed location was decided by the poultry raiser, a male household member, or both together.

Most participants noted positively that their sheds provided birds protection against predators. Both of the VARD beneficiaries and one participant from Khulna had sheds with multiple compartments and felt that the compartments were helpful for housing different types and ages of poultry. Another positive attribute of the sheds mentioned was that they were easy to clean. Intervals for cleaning sheds (i.e., removing poultry feces) ranged from every other day to monthly, with more opting for intervals longer than one week between cleanings. Cleaning the shed was the responsibility of the poultry raiser, though a minority of female participants said that a male household member helped them to clean the shed.

Participants also mentioned problems with their current or previous sheds, such as their condition in inclement weather. A couple of participants said that their sheds were too warm inside during hot weather. Others, like the mud shed, were said to leak, or fall apart during the rainy season. A few participants said that they housed their birds inside the dwelling when it rained because their sheds let water in. Size was another noted issue: all three of the participants with purchased wooden sheds said they were too small; they had between 5 and 7 chickens each, and two also had ducks (2 and 4, each). One poultry raiser with a latrine pit lining ring shed said that it was suitable given the current flock size (two chickens) but that a larger shed would be required if the flock expanded. Another problem reported was that sheds did not always protect against predator attacks: gaps in floors and walls allowed snakes to enter, and wet wood or bamboo was easy for foxes and dogs to break.

When we asked why some poultry raisers kept their poultry in sheds separate from their dwelling, a common response was that poultry needed to be housed outside because of their smell. Participants described poultry and poultry feces as having a smell, sometimes called “gas”, that could cause health problems.

*If I keep the flock inside, then they will defecate inside the house. The feces have a smell and can create problems for people. For that reason, I made a house for them and kept them outside…[the smell] can cause stomach problems…[people] get gastric issues. The children get diarrhea. Even adults can get diarrhea…for that reason [the poultry are] kept like that (outside).*

- Interview #9, Mymensingh Division, Female, 9 chickens and 1 duck

Participants noted that because of predation by foxes, as well as dogs and cats, if birds were kept outside at night, it was necessary to house them in a shed rather than leave them unconfined. A couple of participants noted that in their village one type of predator was more common than others. For a few participants in Dhaka and Rajshahi, the major concern was theft.

*Interviewer: People use different kinds of sheds around the village. Why do you use this kind (a concrete/cement shed)?*

*Participant: If a bamboo shed is used, the thieves lift…the whole thing and steal everything. So, the [shed] we made is mainly used to prevent this issue.*

- Interview #21, Rajshahi Division, Female, 11 chickens

#### Overnight poultry housing inside the household dwelling.

The participants we interviewed who exclusively had overnight poultry housing inside their dwellings were using bamboo cages and barricades. Bamboo cages were either made by a household member or purchased for less than one US dollar. Barricades were made or assembled by household members, including the poultry raiser.

Participants commonly explained that they did not have “space” to keep birds outside. After probing, we understood that this meant either that the household did not have sufficient physical space in the courtyard to build a poultry shed, or that they did not have a shed (i.e., “place”).

In the absence of a shed outside, participants housed birds inside the dwelling due to the risk of predation. However, housing poultry inside the dwelling at night did not mean that predators were not a problem—snakes, rats, and even foxes were still a reported issue.

When we asked about difficulties related to housing poultry inside the dwelling, participants again said that the smell was problematic because it could create health problems, like diarrhea, because it caused general discomfort, or because it disturbed guests.

*I don’t face that many problems. However, there are some issues, such as…the stench of poop and the chicken’s smell. Because of that it is hard to sleep sometimes.*

- Interview #12, Mymensingh Division, Female, 8 chickens

Though smell was cited as a problem, a couple of participants noted the need to tolerate it to raise poultry.

*It would be better if I could keep the birds outside, but I don’t have the ability to build a shed, so I use [housing inside]. I have no other way, whether it smells badly or not. But if I could make a shed, that would be better.*

- Interview #24, Mymensingh Division, Female, 9 chickens and 2 ducks

Not all participants said they were bothered by the smell. A few also mentioned that they kept their poultry housing clean, so smell was not a problem. In contrast to the poultry raisers with sheds, poultry raisers keeping birds inside mostly said they cleaned poultry housing daily. One anticipated benefit for keeping poultry outside was that a shed could be cleaned less often. However, one participant spoke negatively about how people who kept their birds in sheds did not clean it daily, and sometimes did not clean it at all.

When we asked why some people housed poultry outside in sheds, the idea that people were “doing what they could afford to do” was mentioned.

*The way we are raising [poultry], [other] people are doing the same…Everyone does not keep [poultry] in the home. They keep [them] according to their ability; some also keep poultry outside of the living room…Rich people keep poultry separate (outside). We are poor; we cannot keep poultry separate (outside), so we keep them inside.*

- Interview #27, Mymensingh Division, Female, 2 chickens

The majority of participants keeping poultry exclusively inside their dwelling at night said they wanted to build a shed. The participant followed the above discussion about sheds by saying:

*The important thing is that I have a child and I cannot make do without raising poultry (as they give money, eggs, and meat). I need money to build a shed. My children have to study, and we have other expenses. I need money to build a shed, so I need time to build it.*

- Interview #27, Mymensingh Division, Female, 2 chickens

However, one participant, who previously had a corrugated iron shed outside, had moved her poultry back inside when the shed fell apart three years prior. She said that keeping her poultry inside the dwelling was fine for now, as she only had two chickens.

#### Poultry raisers with overnight poultry housing both inside and separate from the household dwelling.

We interviewed poultry raisers from three households that had overnight housing both inside and separate from the dwelling. In one case, the participant housed her chickens in a latrine pit lining ring shed outside but kept her turkeys in a sleeping room inside the dwelling along with goats and other large animals. This setup was designed by the poultry raiser and her husband to protect their larger, more valuable animals from theft.

In another case, the poultry raiser kept her duck in a single-compartment corrugated iron shed outside but kept her chicken and chicks inside the dwelling under an *ugar* and explained that the two could not be housed together because they would fight or spread disease. She said she did not have the funds for two sheds.

In the third case, two poultry raisers in the same household maintained different housing practices: one kept her birds in a shed in the courtyard and the other kept her birds inside the dwelling. The participant who kept her birds inside mentioned that she did not have sufficient physical space outside to house birds, and that she traveled frequently, so chose to keep her poultry inside her dwelling. When we asked the shed-user in the household about the difference between their practices, she explained:

*Participant: I keep my [poultry] outside and the person who made the other [housing inside] doesn’t have space to make a [shed] outside, so, she made [housing] inside the house. Also, the [housing] she made is safe from predators and thieves.*

*Interviewer: Why didn’t you make your [housing] in that way?*

*Participant: Because it smells inside the house and [poultry housing] inside the house doesn’t have proper ventilation. We made our [shed] outside mainly due to hygiene issues. We don’t like to keep the poultry inside the house. It is better to keep the poultry outside.*

*Interviewer: So, you have different views about making [poultry housing].*

*Participant: She made [it] in the way she wants, and we made it the way we prefer. Also…she doesn’t have enough space to [keep them] outside. That is why she…placed [them] inside the house.*

- Interview #21, Rajshahi Division, Female, 11 chickens

#### Exploring preferences, strengths, and weaknesses of different shed designs.

**Fig 4:**
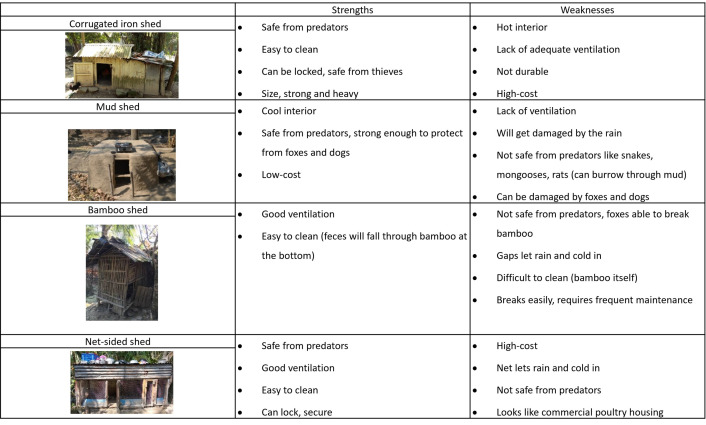
Strengths and weaknesses of different shed designs.

The majority of strengths and weaknesses participants identified for the four distinct shed designs shown during interviews were related to ventilation, weather, and predation, though ease of cleaning, durability, cost, size, and others were also mentioned. (Fig 4) (S3 Data). The most cited strength of the corrugated iron shed was that it would be safe from predators, while the most commonly mentioned weakness was that the interior would get too warm during hot weather. The bamboo shed was said to have good ventilation, but almost all participants said that it was not safe for housing poultry outside at night because predators would either break the bamboo or enter through gaps in the design. The net-sided shed was well-liked by most and seen as having good ventilation and adequate protection from predators, though a couple of participants said negatively that it looked like housing for commercial breeds of poultry rather than *deshi* birds. Several participants had a mud shed previously but had abandoned the design after it had been destroyed by predators or rain. The lack of ventilation in this design was seen as a major weakness, as was the likelihood that it would fall apart due to rain. However, several participants said the mud would keep the interior cool for poultry during hot weather.

Aside from the sheds we showed pictures of, participants mentioned the strengths of other designs, including their own. For example, a couple of participants mentioned that their latrine pit lining ring sheds were considered a durable option, and provided good protection from foxes, and one of the VARD beneficiaries disliked all four of the designs shown because they were all single compartment sheds.

## Discussion

This formative research study provides insights into poultry-housing practices in rural Bangladesh that may contribute to human exposure to poultry and poultry feces, as well as determinants of poultry housing relevant to public health interventions seeking to improve backyard poultry management and biosecurity.

From an exposure perspective, a primary finding from this study is that overnight housing practices can fluctuate across time and seasonally. For examples, housing hens and chicks inside the dwelling overnight for a few weeks was a common practice, even if the household had a poultry shed separate from the dwelling. Keeping hens and chicks together during the first two weeks post-hatching is promoted by development projects to reduce chick death rates [25]. We also found that birds may be kept inside during periods of rainfall, to keep them from getting wet. Varying size and composition of backyard flocks is also a defining characteristic of smallholder poultry production systems [2,55]. However, most studies that explore links between poultry ownership and human health outcomes are cross-sectional, relying on data from a single time point to assess exposure to poultry and poultry feces [11,56]. As such, one-time enumeration of flock composition and housing practices may not reflect average exposure. Future studies should explore risks associated with temporary versus sustained cohabitation with poultry, and assess variables related to poultry ownership and management prospectively and at regular intervals and during different seasons. Our findings, similar to one study in Uganda [57], also suggest that future studies should not solely rely on observation-based methods to record poultry housing presence or location but also ask poultry raisers about their poultry-housing practice (e.g., “Where did you keep your adult chickens last night?”, “Where did you keep your chicks last night?”, “In the last two weeks, how often did you keep your chickens in this shed at night?”).

We found overnight poultry housing in multiple locations within the domestic environment. In accordance with prior research, we differentiated between poultry housing inside versus outside the dwelling [18,19]. However, even when outside, many poultry sheds in our study and others [9,58,59] were still very close to the dwelling (e.g., immediately outside the entryway or on the household’s veranda). In a small intervention-control study of poultry corralling in Peru (n = 55 families), young children in houses with intervention-provided coops or corrals to confine poultry full time (i.e., > 90% of the time) had approximately twice the risk of *Campylobacter*-related diarrhea compared to children in houses without corrals [58]. The authors speculate that corrals, many of which were on the roof or back patio of a house, may have facilitated an accumulation of poultry feces—that, coupled with poor hand hygiene and the possibility that children entered corrals in close proximity to their homes, may have led to greater exposure to *Campylobacter*. In rural Ethiopia, Passarelli et al. (2021) found that having a chicken coop located further from homes was associated with an increased likelihood that children would have visibly clean hands (a proxy for exposure to fecal contamination in the domestic environment) [59]. Classification of poultry housing location beyond a bivariate “inside versus outside”, for example counting the number of steps from the front door of the dwelling to the poultry housing, could lead to better exposure assessment and recommendations for where poultry housing should be located.

Very little of the housing we observed allowed for easy cleaning and removal of poultry feces. Similar to another study in rural Bangladesh [9], we found that poultry housing inside the dwelling was cleaned more frequently than sheds, though we found much wider intervals in between cleanings (>1 week). Indeed, one motivation for housing birds outside the dwelling was to reduce the frequency of cleaning. These findings are important for two reasons. First, good hygiene in poultry housing is important for bird health. Cleaning and disinfection of housing helps to reduce in-flock spread of disease and can therefore play a role in improved poultry health and productivity [25,60]. Next, how poultry housing hygiene impacts human exposure has not been adequately studied. As seen in the previously mentioned corralling intervention in Peru [58], accumulation of poultry feces in poultry housing outside may ultimately increase exposure to the feces. However, daily cleaning of poultry housing inside a dwelling may be equally hazardous. Another consideration is that in this setting and others where women are primarily responsible for cleaning poultry housing, they likely experience a greater exposure risk. Our findings suggest that poultry-housing hygiene practices should also be assessed in future research on human exposure to poultry feces. This area of research would benefit from a One Health perspective, where future studies consider how poultry housing hygiene impacts humans (men, women, children), poultry, and their shared environments (inside the dwelling, in the compound, and in the surrounding environment, and seasonally).

### Determinants of poultry housing type and implications for interventions

**Fig 5:**
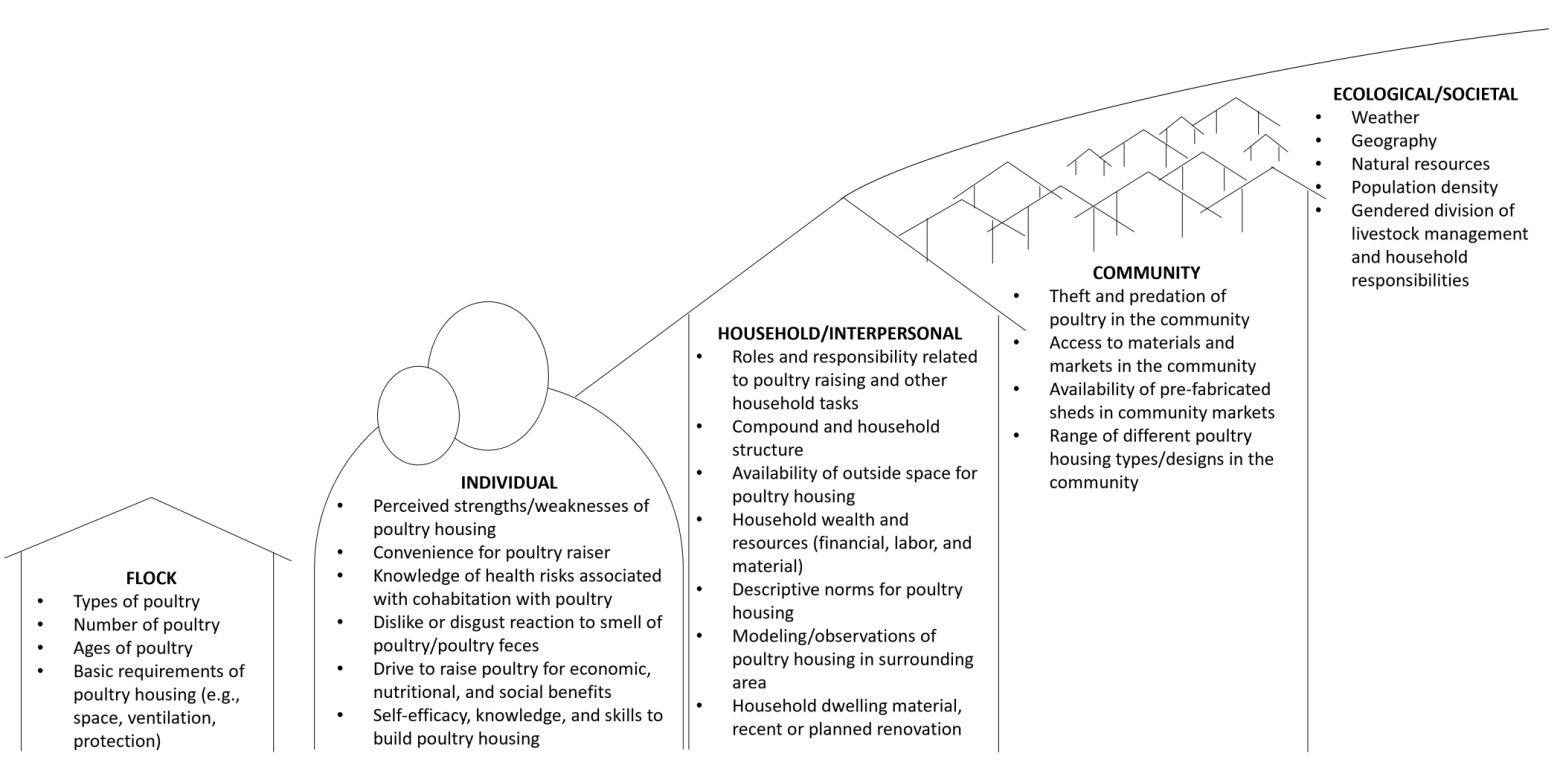
Framework of factors influencing overnight poultry-housing practices in rural Bangladesh.

In Fig 5, we present a framework outlining factors that we identified that influence overnight poultry-housing practices in rural Bangladesh. Our framework takes an ecological perspective and positions poultry management within a multi-level framework [51,61]. The framework also takes guidance from IBM-WASH by acknowledging contextual factors, including background characteristics of the setting and actors, psychosocial factors, such as dislike for the smell of poultry, and technological factors, like ease of use and cost of hardware [51]. We include the poultry flock as a distinct component based on the findings from this study and the literature, which indicate that poultry flock size, composition, and health vary widely, can shape and be shaped by poultry-housing practices, and can influence and be influenced by the poultry raiser, household, community, and larger context in which the poultry raising occurs [1,2,13,55]. Our findings and the framework they generated contain insights for programs across disciplines aiming to improve backyard poultry-housing practices, either to separate poultry from humans or to improve poultry health and productivity. This framework has utility for broader hygiene and One Health interventions: inclusion of the poultry flock as a distinct domain and recognition of environmental factors (e.g., seasonality) will help to better guide studies in the broad scope of factors to consider at the human-animal-environmental interface. This framework should be considered a starting point for future studies; it will need to be adapted and modified based on study setting and objectives.

In this study, interview participants overall demonstrated a preference and willingness to house poultry separate from the dwelling at night. Although the often-cited concerns for predation and theft were present [9,13,37], we found that it was more so the absence of safe poultry housing outside that was driving poultry raisers to keep their birds inside.

While many factors in the framework likely contribute to whether a backyard poultry raiser houses their poultry within their dwelling or separate from it, we recognized several salient facilitators and constraints to having a shed in the courtyard for housing poultry overnight. Whether a poultry-raising household had a shed appeared to be a function of household resources, including availability and access to construction materials, availability and access to skilled labor, available physical space, and area trends. Shed design was driven by the same variables, as well as other contextual factors, like the weather and prevalence of theft and predators. We discuss these determinants below, with implications for public health and livelihood programs.

There were several conditions under which households were more likely to have a shed. First, for many, the materials to build it were readily available. For others, ready-made sheds had been purchased in local markets. We did not explore market availability of sheds across study sites, but field observations suggest that ready-made sheds for chickens and ducks were not available in most locations [Mohammad Rofi Uddin and Shifat Khan, personal communication]. Second, in some villages, most households had poultry sheds separate from the dwelling. This may be reflective of the cost and availability of construction materials in some regions compared to others, or a result of popular practice. Village trends in poultry housing may also be reflective of the social context or a byproduct of other trends. For example, in a village visited for study activities separate from this research, many households had taken out loans to build corrugated iron or *pucca* (brick and plaster) homes, and most households had poultry sheds—likely facilitated by excess materials from home renovations [Mohammad Rofi Uddin, personal communication]. Third, most of our participants’ sheds had been built by a skilled laborer or male household member who possessed the required skills and tools. This finding aligns with studies from Benin, which found that male poultry raisers were more likely to have henhouses—the authors note that this was likely due to their greater access to financial and labor resources (including building housing themselves) [62,63].

On the other hand, lack of finances, materials, and space were noted constraints to building a shed. Guidance on small-scale poultry production in LMICs from the Food and Agricultural Organization (FAO) and similar entities suggests that poultry housing can be made at low-cost by using local materials, such as thatch grass or tree branches [13,25]. However, in some settings, like ours, access to lower-cost building materials in the natural environment (e.g., bamboo) may be limited. In addition, sheds made from low-cost materials may not be seen as safe from predators, thieves, or the weather (e.g., mud sheds). Lastly, for households with limited space in their compound, building a shed could prove difficult. One recommendation from the Australian Centre for International Agricultural Research (ACIAR) is that overnight housing for 10–15 birds should be one meter wide by one or two meters long [13]. Compounds with multiple poultry-raising households are unlikely to have the space to be able to build poultry housing within this recommendation. In this study, we documented several multi-story or multi-compartment sheds, which could be one solution to the problem of limited physical space.

Aside from having a shed, factors outlined in Fig 5 also contributed to whether a household actually kept their poultry in the shed overnight. Our findings suggest that weather, predation, theft, size and composition of flock, and attributes of the shed, like the number of compartments, factor in to if poultry will be kept within the dwelling or a shed overnight.

Several of the factors driving birds to be kept within the dwelling overnight could be assuaged by housing with adequate ventilation, protection from predators, and multiple compartments. However, in this setting, we would not recommend infrastructure provision alone: programs that promote a one-size-fits-all approach to poultry housing and fail to consider the importance of personal preferences, variations in availability and access to materials, flock size and composition, and other contextual factors may face poor uptake and limited use. We make three main recommendations for programs intervening in this space. First, programs could emphasize the features of sheds that maximize both the health and safety of birds and the separation between poultry and humans. For example, to facilitate moving all birds outside the dwelling, programs might consider promoting shed designs that have protected spaces for laying hens and chicks and are large enough to accommodate normal fluctuations in flock size. The Food and Agricultural Approaches to Reducing Malnutrition cluster‐randomized trial in Sylhet division, Bangladesh took a similar approach and saw increases in shed ownership and use [64], and a community-based backyard poultry intervention in Benin also advocated for allowing poultry raisers to adapt a general set of recommended shed features as needed [65]. Second, programs should assess which materials are available, affordable, and acceptable for poultry housing in hyper-local contexts, and work with poultry raisers to generate feasible and acceptable recommendations for constructing low-cost, safe, and durable poultry housing. Third, in contexts where women are the primary poultry raiser but not traditionally involved in building or construction projects, programs will need to consider existing gender roles and encourage men to provide the resources needed to improve poultry management, without limiting women’s control over their flock, or provide women with the skills needed to build and maintain poultry housing.

The findings from this study informed a backyard poultry management intervention in rural Bangladesh with the goal of separating young children from poultry and poultry feces. Following the guidance above, households (female poultry raisers and male household members) participated in a behavior change communication and counseling intervention that included a recommendation to build an improved shed for nighttime poultry housing; the intervention recommended that sheds be outdoors, have multiple compartments and cross-ventilation, and be elevated off the ground. Half of the households also received a subsidy for construction costs of the shed [41].

There are some limitations to this research. First, while we conducted transect walks in all divisions of Bangladesh to add representative descriptive data to the knowledge base of poultry-housing practices across the country, there is likely additional variation in practices beyond what was documented in this study. In addition, given that documenting the prevalence of housing practices was not a study objective, not all poultry housing observed during transect walks was photographed and not all housing practices (e.g., use of shed) were catalogued; this limits our ability to report on the frequency of housing archetypes within and across divisions. Another limitation is that we only conducted semi-structured interviews in a selection of communities visited during transect walks, and all interviews with poultry raisers exclusively keeping birds inside the dwelling overnight were conducted in one division (though in multiple villages). Nevertheless, our findings are reflective of other studies in Bangladesh and similar settings [9,55,59,66,67], and suggest that the high-level observations made here are relevant to backyard poultry production systems both in Bangladesh and elsewhere. Second, we only interviewed two men (both poultry raisers). Our findings suggest that men can play a determinant role in poultry-housing practices, even if their other contributions to poultry raising are limited. Interviewing additional men could have helped to triangulate findings and further explore constraints and facilitators related to construction of poultry housing separate from the dwelling. For this reason, we purposefully interviewed male household members in later stages of this research. Another limitation is that we did not explore all types of housing during interviews, such as cages or barricades in storage rooms or cowsheds. Exploring the determinants of shared housing between poultry and other domestic animals could provide valuable insight for interventions aiming to reduce the introduction and spread of disease among domesticated animals, and between domesticated animals and humans.

## Conclusion

This study was conducted to inform an intervention to limit young children’s exposure to poultry and poultry feces in rural Bangladesh. First, we documented overnight poultry-housing practices and a range of different designs, materials, and locations of the housing. Then, we explored factors that influenced where and how poultry raisers keep their poultry at night. We recommend that future studies measuring human exposure to poultry and poultry feces assess exposure prospectively and at regular intervals to capture variations in housing practice, and include assessments of poultry-housing hygiene practices. In this study, we found that poultry raisers generally preferred and were willing to house their birds in sheds outside. Poultry housing location and type was influenced by multiple, context-specific factors, including flock size and composition, household resources and dynamics, individual psychosocial factors, and background characteristics of the study setting. These findings contain insights for programs aiming to improve backyard poultry management and biosecurity, including the need to consider what is feasible and acceptable for poultry housing in hyper-local contexts and the differing roles that women and men may hold in constructing and managing poultry housing.

## Supporting information

S1 Data Primary and secondary materials of sheds in each division. (XLS)

S2 Data Interview participant characteristics and poultry housing types. (XLSX)

S3 Data Strengths and weaknesses of shed designs. (XLSX)
